# How to improve public environmental health by facilitating metro usage on weekend: exploring the non-linear and threshold impacts of the built environment

**DOI:** 10.3389/fpubh.2024.1469578

**Published:** 2024-11-04

**Authors:** Bozhezi Peng, Tao Wang, Yi Zhang, Chaoyang Li

**Affiliations:** State Key Laboratory of Ocean Engineering, School of Ocean and Civil Engineering, Shanghai Jiao Tong University, Shanghai, China

**Keywords:** built environment, metro ridership, machine learning, nonlinearity, public environmental health

## Abstract

**Introduction:**

The accelerated motorization has brought a series of environmental concerns and damaged public environmental health by causing severe air and noise pollution. The advocate of urban rail transit system such as metro is effective to reduce the private car dependence and alleviate associated environmental outcomes. Meanwhile, the increased metro usage can also benefit public and individual health by facilitating physical activities such as walking or cycling to the metro station. Therefore, promoting metro usage by discovering the nonlinear associations between the built environment and metro ridership is critical for the government to benefit public health, while most studies ignored the non-linear and threshold effects of built environment on weekend metro usage.

**Method:**

Using multi-source datasets in Shanghai, this study applies Gradient Boosting Decision Trees (GBDT), a nonlinear machine learning approach to estimate the non-linear and threshold effects of the built environment on weekend metro ridership.

**Results:**

Results show that land use mixture, distance to CBD, number of bus line, employment density and rooftop density are top five most important variables by both relative importance analysis and Shapley additive explanations (SHAP) values. Employment density and distance to city center are top five important variables by feature importance. According to the Partial Dependence Plots (PDPs), every built environment variable shows non-linear impacts on weekend metro ridership, while most of them have certain effective ranges to facilitate the metro usage. Maximum weekend ridership occurs when land use mixture entropy index is less than 0.7, number of bus lines reaches 35, rooftop density reaches 0.25, and number of bus stops reaches 10.

**Implication:**

Research findings can not only help government the non-linear and threshold effects of the built environment in planning practice, but also benefit public health by providing practical guidance for policymakers to increase weekend metro usage with station-level built environment optimization.

## 1 Introduction

The accelerated urbanization and motorization have brought severe environmental challenges, including air pollution, traffic congestion and climate change (1). The air and noise pollution brought by car dependence are the critical threats to public health, which can damage the physical and mental health of people (2). Under this circumstance, transit-oriented development (TOD) has been advocated among many countries to promote urban rail transit system (3). The large-scale construction of metro system has shifted from developed countries to developing contexts (4). In China, the metro system has been constructed in 59 cities and the total length has reached 11232.65 km by the end of 2023 (5). Since the urban traffic carbon emission is a critical reason of climate change, it is imperative for urban planners to improve public environmental health by facilitating metro usage.

Due to the large capacity, low cost and travel time reliability, metro system has become an effective transport alternative for not only the commuting trips on weekdays but also the leisure trips on weekends (1). The trip purpose and travel behavior of metro users can be significantly different between weekdays and weekends (6). For example, there are more commuting trips on weekdays with obvious rush hours, while more entertaining trips with no obvious peak hours on weekends (7). Since the travel modes for commuting people are relatively fixed, promoting metro usage on weekend can not only mitigate traffic congestion and reduce carbon emissions, but also benefit public health from multiple perspectives.

Compared to other factors which may influence the metro ridership (e.g., weather, fare, etc.), the built environment is more suitable to optimize at different metro stations (8). With the development of geographic information systems (GIS) and availability of big data, direct ridership models (DRMs) become more popular in recent metro ridership literature (9). Many of DRMs derived from ordinary least squares (OLS) regression (10), multilevel regression (11), or geographically weighted regression (12), by assuming a linear or loglinear relationship. However, the nonlinearity between the built environment and metro usage has been recently investigated by different DRMs based on several machine learning algorithms, such as Gradient Boosting Decision Tree (GBDT), Random Forest (RF), eXtreme Gradient Boosting (XGBoost) and Light Gradient Boosting Machine (LightGBM) (4, 13, 14). Moreover, the non-linear influences of built environment have been discovered on different travel behavior, including shared bikes (15), shared e-scooters (16), ride-splitting (17), driving distance (18) and ride-sourcing (19). Among these emerging non-linear studies on metro ridership, most of them focused on non-linear effects of the built environment on weekday metro ridership (4, 13, 14), ignoring the temporal heterogeneity on weekend metro usage. The non-linear associations between built environment and metro ridership can be quite diverse between weekdays and weekends, while previous studies failed to address this issue.

To fill the gap, this study aims to promote the metro usage and improve the public environmental health by discovering the non-linear impacts of built environment on weekend metro usage. By utilizing various datasets and GBDT approach, this study attempts to address two research questions: (1) What is the relative importance of each built environment variable in affecting weekend metro ridership? (2) Does the built environment show non-linear impacts on weekend metro usage? What are the threshold and effective ranges?

The remaining part of this paper is structured as follows. Next section reviews the studies on associations between the built environment and metro ridership. Section three introduces the data, variables and methodology. Section four concludes the results. Section five discusses the research findings. The last section summarizes the paper and points out the limitation.

## 2 Literature review

Due to the popularity of urban rail transit system and transit-oriented development, studies on impacts of built environment on metro usage have brought increasing attentions in the past few decades (4, 13, 14, 20). In the past few years, DRMs have become popular than traditional ridership prediction model because of the convenience of data collection (13).

Although scholars have evaluated the built environment from different aspects, most measured the built environment by “5Ds,” including density, diversity, design, destination accessibility and distance to transit (21). Higher activity density can increase the possibility of using metro system. For example, population density or employment density have significant and positive impacts on metro usage (22), both in developed countries (23) and developing countries (24–26). However, recent studies employed GBDT model and proposed that non-linear impacts of density on metro usage may appear negligible if it beyond certain threshold (4, 13, 14, 20).

Diversity, such as mixed land use, can improve metro usage by making the metro station surroundings more appealing. For example, land use mixture has been explored to have positive impacts on metro ridership in Spain, South Korea and China (12, 27, 28), but studies in other countries show insignificant effects of land use mix (23, 29–31). Recently, the non-linear impacts of diversity on metro ridership has been found to be non-trivial only when they are within certain ranges (13, 14).

Design measures road network within the station area. Design features, including street or intersection density can show positive impacts (14, 23, 24, 32–34) or negative effects on metro ridership (26, 28, 35). Recently, studies on DRMs have pointed out that design features have positive impacts on metro usage only if they are in certain ranges (13, 14).

Destination accessibility measures the accessibility to certain areas (city center) or facilities (shopping center). For example, some studies examined the non-linear associations between distance to city center and metro ridership (4, 13). However, the impacts of distance to city center are found to be insignificant in other studies (24, 34). Distance to transit, including bus stop and bus route, have also been explored by studies in different contexts (14, 20, 36, 37).

To sum up, some studies have used DRMs to analyze the impacts of built environment on metro usage, but almost neglect the metro usage on weekends. For these gaps, this study tries to improve the public environmental health by discovering the non-linear impacts of built environment on weekend metro usage.

## 3 Materials and methods

### 3.1 Study area

**Figure 1:**
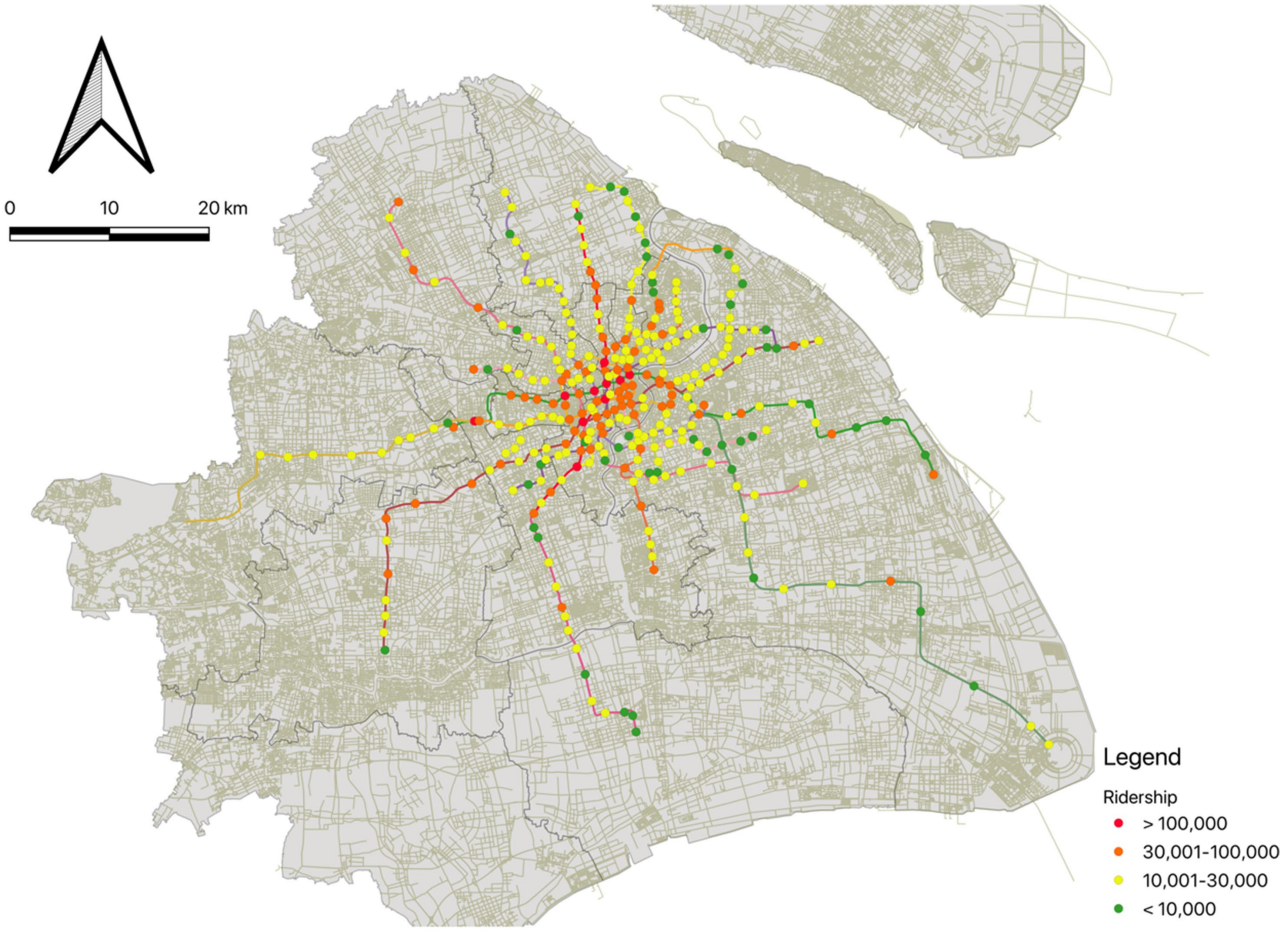
Weekend metro ridership of 328 metro stations in Shanghai.

In this study, we utilized 1 month smartcard data on May 2023, including 17 lines and 328 stations in Shanghai (Figure 1). The smartcard data was provided by the Shanghai Government Data Portal^1^ with hourly passengers. The raw smartcard data included number of hourly inbound and outbound passengers for each metro station. Daily ridership on weekends of each station was then aggregated by adding up hourly inbound and outbound passengers. The average station ridership on weekends is 27,259 riders per day. People’s Square Station, the interchange station of three lines which located at the city center, has the highest ridership of 243,938 passengers. Thirty-two stations have more than 50,000 riders per day, while 55 stations have fewer than 10,000 daily ridership.

### 3.2 Variables

Twelve built environment variables were included to measure 5Ds built environment in this study. Multiple sources and platforms are used to collected the data of the built environment characteristics, including OpenStreetMap and AMAP API.

#### 3.2.1 Density

Population density around metro station is calculated within 500 m buffer, based on the WorldPop population data with 100 m resolution. Using POI data from AMAP API, employment density is determined by the percentage of employment-related point of interests within 500 m buffer. Rooftop density, the measure of land development, was also calculated within 500 m buffer based on the rooftop area datasets (38).

#### 3.2.2 Diversity

Land use mix, the entropy index for different land use, was utilized to measure station level land use diversity. Since the land use data was not open access to the public, 23 categories of point of interests are used as the alternative to calculate the land use mix entropy within 500 m buffer, including catering, shopping, education, employment, entertainment, tourism, public service, sports, green space, etc.

#### 3.2.3 Design

Intersection and road density were used to measure street design, based on the data from OpenStreetMap. Road density was measured by removing highways and sidewalks from the OpenStreetMap street network, while number of intersections is measured by counting 3-way or more intersections.

#### 3.2.4 Destination accessibility

Network distance to CBD and straight distance to the nearest Sub-CBD were involved to measure the effects of accessibility. The CBD and several city Sub-CBDs were chosen according to the official document by Shanghai government (39). Network distance to the nearest highway entrance is was also used to assess the destination accessibility.

#### 3.2.5 Distance to transit

Bus stop and bus line are counted within the station service area, while straight distance to the nearest bus stop is selected to measure the distance to transit.

**Figure 2:**
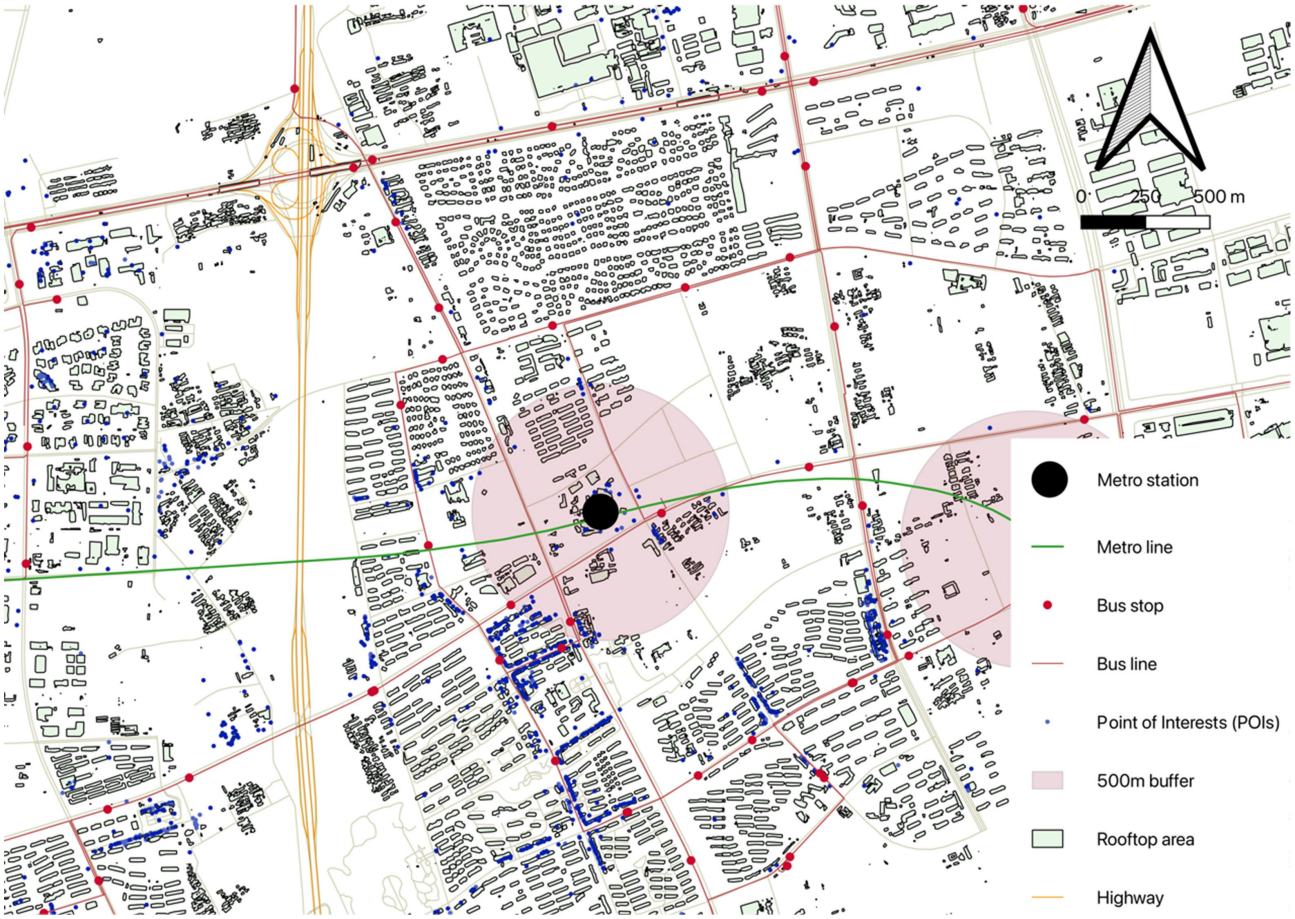
An example of built environment variables within station area.

All the built environment characteristics are measured within 500 m buffer by QGIS (Figure 2). Table 1 summarizes the statistics of all built environment variables.

**Table 1:** Statistics of all variables.

Variables	Description	Mean	S.D.	Min	Max	Data source
Dependent variable						
Weekend ridership	Daily metro ridership on weekends (count)	27,259	27,839	1,011	243,938	Metro smartcard data of Shanghai on May 2023
Built environment variables						
Density						
Population density	Population density within 500 m buffer (1,000 people/km^2^)	16.83	10.52	0.04	41.58	WorldPop population data 2023
Employment density	Ratio of employment POI within 500 m buffer	0.14	0.15	0.03	0.95	Point-of-interest (POI) data 2023
Rooftop density	Rooftop area ratio within 500 m buffer	0.18	0.06	0.02	0.45	Vectorized rooftop area data 2020
Diversity						
Land use mixture	The entropy index $\frac{-\sum_{i=1}^{m}\left(p_{i}\right)\ln\left(p_{i}\right)}{\ln\left(m\right)}$ where m denotes different POI and $p_{i}$ represents the ratio.	0.72	0.13	0.14	0.87	Point-of-interest (POI) data 2023
Design						
Road density	Road centerline length per km^2^ (km/km^2^)	5.83	1.94	1.22	13.84	OpenStreetMap data 2023
Intersection	Number of intersections within 500 m buffer (count)	9.24	6.12	0.00	42.00	OpenStreetMap data 2023
Destination accessibility						
CBD	Network distance to CBD (km)	14.14	10.29	0.16	65.90	OpenStreetMap data 2023
Sub-CBD	Straight distance to the nearest Sub-CBD (km)	6.75	5.22	0.00	37.85	OpenStreetMap data 2023
Highway	Network distance to the nearest highway (km)	1.25	1.46	0.04	6.62	OpenStreetMap data 2023
Distance to transit						
Bus stop	Number of bus stops within 500 m buffer (count)	6.42	3.50	1.00	26.00	Point-of-interest (POI) data 2023
Bus line	Number of bus routes within 500 m buffer (count)	17.15	10.27	0.00	62.00	Point-of-interest (POI) data 2023
Nearest bus stop	Straight distance to the nearest bus stop (km)	0.12	0.07	0.01	0.42	Point-of-interest (POI) data 2023

### 3.3 Methodology

We employ Gradient Boosting Decision Tree (GBDT) approach to analyze the non-linear effects of the built environment on weekend metro usage. GBDT has several merits for this study. GBDT do not pre-assume linear association between different variables (40). It can also visualize the non-linear relationship by depicting partial dependent plot, which shows the marginal effect on the predictions (41). Meanwhile, GBDT helps to evaluate the contribution of each feature by automatically calculating feature importance (14). Moreover, GBDT is not sensitive to multicollinearity problems, which makes it possible to examine non-linear impacts of different features on weekend metro usage, even they are highly correlated. The effectiveness of GBDT has been recently proved by several studies to evaluate the non-linear effects of built environment on different kinds of travel behavior.

Mathematically, GBDT sets the approximation function $F_{T}\left(x\right)$ by combined several decision trees, and aims to minimize the loss function $L\left[y,F\left(x\right)\right]={\left[y-F\left(x\right)\right]}^{2}$. The approximation function $F_{T}\left(x\right)$ is given by Equation 1:


(1) $$F_{T}\left(x\right)=\overset{T}{\underset{t=1}{\sum }}f_{t}\left(x\right)=\overset{T}{\underset{t=1}{\sum }}\theta_{t}h\left(x;\eta_{t}\right)$$

where T is number of trees, $\eta_{t}$ is the parameter of the $t^{th}$ tree $h\left(x;\eta_{t}\right)$, $\theta_{t}$ is the weight of $h\left(x;\eta_{t}\right)$ which can be calculated by minimizing the loss function. The optimization process includes several iterative steps. First, the initialization function is determined as Equation 2:


(2) $$f_{0}\left(x\right)={\mathrm{argmin}}_{\theta }\overset{N}{\underset{i=1}{\sum }}L\left(y_{i},\theta \right)$$

Second, the residual error $r_{t,i}$ is derived for each sample i in $t^{th}$ iteration as Equation 3:


(3) $$r_{t,i}=-{\left[\frac{\partial L(y_{i},f\left(x_{i}\right)}{\partial f\left(x_{i}\right)}\right]}_{f\left(x\right)=f_{t-1}\left(x\right)}$$

Third, ($x_{i},r_{t,i})$ are utilized to fit the $t^{th}$ $\left(t=1,2,\ldots ,T\right)$ tree $h\left(x;\eta_{t}\right)$ by getting the $R_{t,j},\left(j=1,2,\ldots ,J_{t}\right)$, while $J_{t}$ is the tree size. After that, we can use tree traversal to determine the optimal gradient as Equation 4:


(4) $$\theta_{t}={\mathrm{argmin}}_{\theta }\overset{N}{\underset{i=1}{\sum }}L\left(y_{i},F_{t-1}\left(x_{i}\right)+\theta h\left(x;\eta_{t}\right)\right)$$

Thus, we can rewrite the iterative equation as Equation 5:


(5) $$f_{t}\left(x\right)=f_{t-1}\left(x\right)+\theta_{t}h\left(x;\eta_{t}\right)$$

To moderate overfitting, learning rate is proposed as the shrinkage parameter$\varepsilon \;\left(0<\varepsilon \leq 1\right)$ (41). Therefore, the final function could be written as Equation 6:


(6) $$F\left(x\right)=f_{t}\left(x\right)=f_{t-1}\left(x\right)+\varepsilon \;\theta_{t}h\left(x;\eta_{t}\right)$$

For each feature, the feature importance can be calculated by the final model. The importance of feature $x_{i}$ can be determined as Equation 7 (42):


(7) $$I_{x_{i}}=\sqrt{\frac{1}{T}\overset{T}{\underset{t=1}{\sum }}\overset{J_{t}}{\underset{j=1}{\sum }}d_{j}}$$

where j denotes tree nodes, and $d_{j}$ refers the differences of loss function when make $j^{th}$ tree splitting.

Mathematically, the partial dependence of an independent variable $x_{s}$ can be calculated as Equation 8 (43):


(8) $$F_{s}\left(x_{s}\right)=E_{x_{c}}\left[F\left(x_{s},x_{c}\right)\right]$$

where $x_{c}$ represents other variables. Then, the partial function $F_{s}\left(x_{s}\right)$ can be determined by averaging over all samples as Equation 9:


(9) $$\bar{F_{s}}\left(x_{s}\right)=\frac{1}{N}\overset{N}{\underset{i=1}{\sum }}F\left(x_{s},x_{c}\right)$$

Shapley additive explanations (SHAP) can also interpret the model outputs by machine learning models (44). Shapley value (45) are used in SHAP to evaluate the effects of each variable as Equation 10 (44):


(10) $$\varphi_{v}=\underset{z^{\prime }\subseteq x^{\prime }}{\sum }\frac{|z^{\prime }|!\left(V-|z^{\prime }|-1\right)!}{V!}\left[f_{x}\left(z^{\prime }\right)-f_{x}\left(z^{\prime }/V\right)\right]$$

where V denotes number of variables, $\varphi_{v}$ represents the contribution of variable v, $f\left(x\right)$ refers model outputs, $\left|z^{\prime }\right|$ counts non-zero entries in $z^{\prime }$.

However, GBDT does have certain restrictions. For example, it cannot perform significance tests and produce coefficient of variables, while feature importance can be used as the substitution. It is also easy to overfit, while using cross-validation and suitable shrinkage parameter can solve this problem (41). In this study, we conduct 5-fold cross-validation and selected the learning rate as 0.001. We get the optimal GBDT model with the lowest RMSE after 2,893 iterations, and the pseudo-*R*^2^ is 0.83.

## 4 Results

### 4.1 Feature importance of the built environment

Table 2 presents the relative feature importance and ranking in determining metro usage on weekends. Land use mixture has the largest predictive power, with the relative importance of 16.26%. As the measurement of diversity, it has been observed as a critical factor on metro ridership prediction by many previous studies in different contexts (4, 13, 28, 33). Distance to CBD, a measure of regional accessibility, has the second large relative importance, with a contribution of 13.61%. Other destination accessibility variables, including distance to highway (7.21%) and distance to Sub-CBD (5.21%), also have non-trivial impacts on weekend metro usage. The importance of bus line is also substantial, accounting for 12.17% and ranking 3rd over all independent variables. This corresponds to the existing findings that distance to transit can notably affect the metro usage (4, 13). Among five categories of built environment, density features have the largest relative importance of 29.29% on ridership prediction, collectively contributed by three density variables. By contrast, design variables (e.g., intersection and street density) only shown trivial impacts on weekend metro usage, with relative importance of only 2.78 and 2.61%, respectively.

**Table 2:** Relative importance and ranking of variables.

Category	Features	Ranking	Relative importance
Density (29.29%)	Population density	6	9.18%
	Employment density	4	10.06%
	Rooftop density	5	10.05%
Diversity (16.26%)	Land use mixture	1	16.26%
Design (5.39%)	Road density	12	2.61%
	Intersection	11	2.78%
Destination accessibility (26.03%)	CBD	2	13.61%
	Sub-CBD	9	5.21%
	Highway	7	7.21%
Distance to transit (23.03%)	Bus stop	8	7.06%
	Bus line	3	12.16%
	Nearest bus stop	10	3.81%

### 4.2 SHAP beeswarm plot of the built environment

To discover the contribution of each variable and analyze how variables of stations influence the metro usage, SHAP beeswarm plots (also called the SHAP summary plots) are employed in this study.

**Figure 3:**
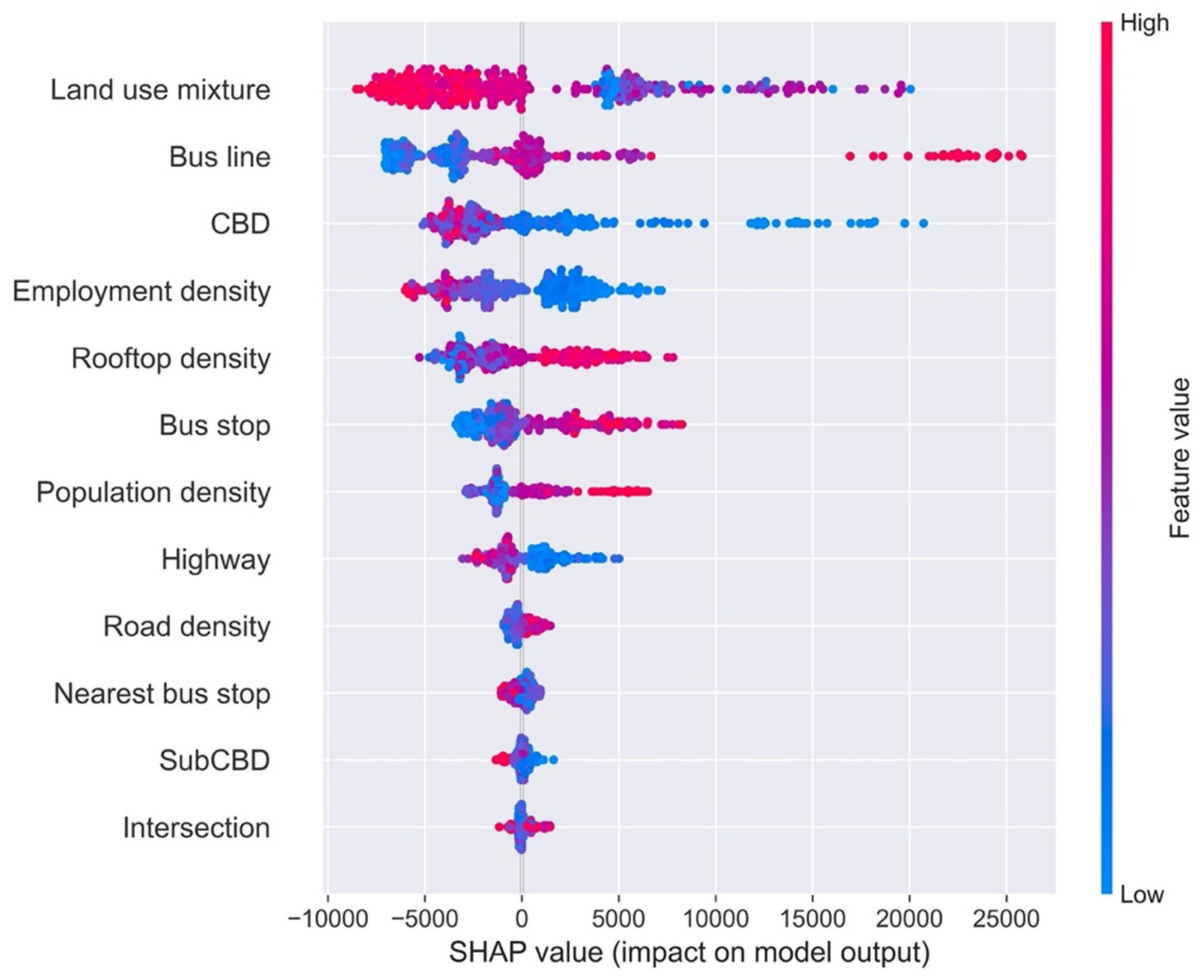
SHAP beeswarm plot of built environment.

The SHAP beeswarm plot sorts variables by mean absolute value of SHAP values, while uses SHAP value to show the effect distribution of variables (Figure 3). Each station is displayed by one point for each variable, while the horizontal axis presents SHAP values. Value of each feature is shown in different colors.

As shown in Figure 3, land use mixture ranks first by SHAP values, which is similar to the feature importance results. Meanwhile, Number of bus line, distance to CBD, employment and rooftop area are other top five significant variables, which is same with the feature importance results but with little difference with ranking. Moreover, number of bus stops, which ranked only 8th by relative importance, are the 6th most significant variable by SHAP values.

Bus line, rooftop density, bus stop and population density are positively related with SHAP value, while land use mixture, CBD and employment density show negative associations. It means that large number of bus lines and bus stops, high rooftop density and population density (in red color) can increase more metro ridership on weekends, while high land use diversity, long distance to CBD and high employment density (in red color) lower the weekend metro ridership.

### 4.3 Non-linear impacts of built environment on weekend metro usage

**Figure 4:**
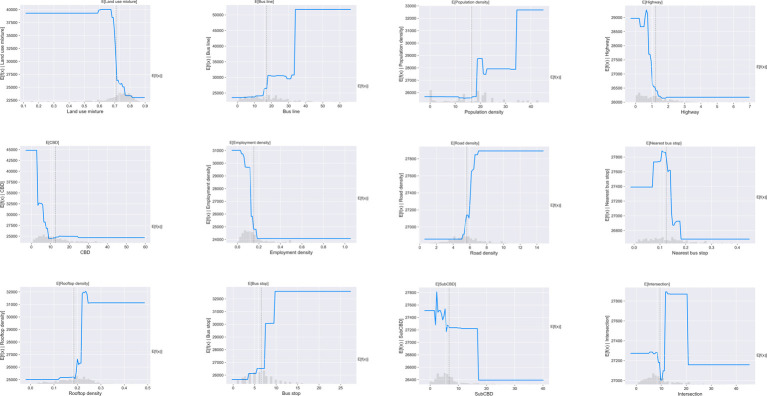
Partial dependence plots on weekend metro usage.

To explore the relationship between the built environment and weekend metro ridership, partial dependence plots (PDPs) are employed in this study. Overall, all independent variables shown non-linear associations with weekend metro usage. Figure 4 presents the non-linear impacts of built environment variables on weekend metro usage.

As shown in Figure 4, the weekend metro ridership remains (at about 40,000) when land use mixture entropy is smaller than 0.65. However, the weekend metro ridership drops substantially to less than 25,000 when the entropy moves from 0.65 to 0.75, and no further decrease occurs.

Bus line is positively associated with weekend metro usage. The metro usage keeps stable at less than 25,000 when bus route is less than 15. After that, the ridership suddenly increases to 30,000 as bus route moves from 15 to 20, and no increase in metro ridership has been found when bus line is between 20 and 30. However, the weekend metro ridership sharply increases from 30,000 to 50,000 when number of bus line reaches 35, and then remain constant.

The association between distance to CBD and weekend metro ridership is negative. The weekend ridership drops dramatically from 45,000 to 25,000 when the distance to CBD grows from 0 to 10 km. However, no further decrease of metro ridership has been found when the distance to CBD exceeds 10 km. Similar pattern has been found for distance to Sub-CBD.

Rooftop density has positive effects on weekend metro ridership. When the rooftop density is less than 0.2, metro ridership keeps 25,000. After that, the weekend ridership rises substantially from 25,000 to 31,000 when rooftop density between 0.2 and 0.25. As shown in the PDPs, as bus stop increases from 0 to 10, weekend metro ridership rises by 6,000. However, this effect looks negligible when there are more than 10 bus stops.

Meanwhile, the distance between metro station and nearest transit station has negative impacts to weekend metro usage, with an effective interval of 100–200 m. However, this effect is limited and the difference in metro ridership is only about 1,500, echoing the small relative importance of this variable in metro ridership prediction.

**Figure 5:**
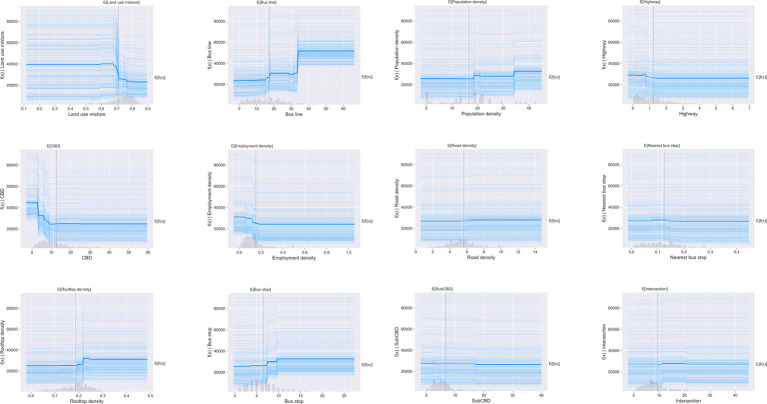
Combination of PDPs and ICEs of built environment on metro usage.

Overall, PDPs show the average effect of the built environment variables without specific instances. To visualize the partial dependence of one variable on weekend metro ridership for each station, we also combined Individual Conditional Expectation (ICE) curves with PDPs as shown in Figure 5. In Figure 5, the ICE curves are presented in light blue lines, while the PDP is shown in dark blue line as the average.

As shown in Figure 5, for each independent variable, all the ICE curves seem to follow the similar pattern with the partial dependence plot. It means that there is no obvious heterogeneous relationship created by interactions. Under this circumstance, employing PDPs in this study can provide good summary of the impacts of built environment on predicted metro usage on weekends.

## 5 Discussion

Promoting metro usage on weekends by optimizing station-level built environment is a critical way to address a series of environmental challenges from accelerated urbanization and mobilization. This study employed GBDT approach to evaluate the non-linear associations between the built environment and weekend metro ridership in Shanghai. Several model interpretation methods are utilized to unravel the non-linear impacts of factors on weekend metro usage.

Based on the results of relative importance and SHAP values, we recognized that land use mixture, the distance to CBD and number of bus lines are three most important factors on affecting weekend metro usage.

Among these three factors, land use mixture and distance to CBD are found to be negative associated with weekend metro ridership, while number of bus lines is found to be positive related with weekend metro usage. Higher land use mixture usually represents more average land use types within the station catchment area. However, many metro users take metro on weekend for a specific purpose (e.g., shopping, food, or tourism), and stations with relatively lower land use mixture may thus have more metro riders. It is intuitive that distance to CBD is negative related with weekend metro usage. Due to the traffic jam and shortage of parking spaces within CBD area in megacities like Shanghai, driving to the CBD on weekends may not as convenient as taking the metro. Therefore, metro stations which are close to the CBD can attract more metro users during the weekend. More bus lines near the metro station can provide sufficient first/last-mile services for metro users to access the metro station on weekend, which enlarge the station catchment area and facilitate weekend metro usage.

Based on the results of partial dependence plots, all the built environment variables show non-linear impacts on weekend metro usage with certain threshold and effective ranges (Table 3). Weekend metro ridership shows a significant decrease when land use mixture moves from 0.65 to 0.75. The complex relationship between land-use diversity and weekday or weekend metro usage is also found by many literature in different contexts (4, 14, 20).

**Table 3:** Effective ranges of variables.

Variables	Effective range/Threshold	Association
Land use mixture	0.65–0.75 (scale)	Negative
Bus line	15–20, 30–35 (count)	Positive
CBD	2–10 (km)	Negative
Employment density	0–0.2 (scale)	Negative
Rooftop density	0.2–0.25 (scale)	Positive
Bus stop	3–10 (count)	Positive
Road density	5–7 (km/km^2^)	Positive
Highway	0.5–2 (km)	Negative

The distance to CBD is negatively related with weekend metro usage between 2 to 10 km, which seems to be reasonable that metro stations near city center may have densified population and thus more metro passengers. The distance to CBD has no significant impact on weekend metro usage when it is beyond this range. The sharp rise of weekend metro usage has been found when number of bus lines increases from 15 to 20 and 30 to 35, while the ridership remains nearly constant when number of bus lines is within other ranges. Existing literature has also suggested the positive impacts of bus lines on metro usage, while the impacts can be mediated if bus route is more than 40 (4). Rooftop area has a positive association with weekend metro ridership, with a dramatic rise between 0.20 and 0.25. This indicates that high level of land use development can facilitate the weekend metro usage, but excessive development may have trivial effects on further increase. Number of bus stops has positive effects on weekend metro ridership, with an effective range between 3 and 10. Similar threshold impacts of bus stops are found in different cities but with different thresholds (13, 14).

## 6 Conclusion

To improve the public environmental health by facilitating metro usage on weekend, this study employed GBDT approach to evaluate the non-linear and threshold impacts of the built environment on weekend metro ridership. Compared to conventional models with linear presumption, investigating the non-linear effects help policymakers and urban planners recognize the thresholds and effective ranges of the built environment characteristics, which can benefit public health by making customized strategies and policy interventions. The empirical finding may contribute threefold to the existing studies.

First, this study estimates the feature importance of built environment characteristics in predicting weekend metro ridership. According to the results, the top-five variables with highest importance are land use mixture (16.26%), distance to CBD (13.61%), bus line (12.17%), employment density (10.06%) and rooftop density (10.05%). Results can help urban planners identify the role of different built environment characteristic and issue differentiation strategies.

Second, it depicts SHAP beeswarm plot to show the impact of each variable on the prediction. The top-5 important variables by SHAP beeswarm plot are same to relative importance. Bus line, rooftop density, bus stop and population density are positively related with SHAP value, while land use mixture, distance to CBD and employment density are negativity associated with SHAP value. Therefore, urban designers should pay different attention to the built environment characteristics to promote metro usage.

Third, we depict the non-linear impacts of the built environment by combining PDPs with ICEs. Most variables have obvious thresholds on determining weekend metro ridership. Results show that maximum weekend ridership occurs when land use mixture entropy is smaller than 0.7, number of bus lines reaches 35, rooftop density reaches 0.25, and number of bus stops reaches 10. The non-linear relationship and their effective ranges help policymakers increase metro ridership on weekends by optimizing station-level land use.

Several limitations merit further study. First, the influences of built environment features on weekend metro usage may vary in different contexts. Therefore, relevant studies are encouraged to explore or validate the non-linear associations between the built environment and weekend metro ridership. Second, this study uses the 500 m buffer for most independent variables, while 400 m buffer (13) and 800 m (20) are used by different station-level built environment studies. Because the real service area of metro stations may vary in different cities and stations, future studies are welcome to testify the results with different buffer zones. Third, we only explore the effects of a limited number of built environment characteristics. With the development of big data and GIS, more comprehensive built environment attributes (e.g., number of parking spaces, demographics, sidewalk density) with finer data are welcomed for further exploration in different contexts. Fourth, PDPs may be misguided when independent variables are correlated with each other (e.g., bus stop and bus line), while accumulated local effects (ALE) plots can be used as an unbiased alternative to address the multicollinearity issue in further studies. Fifth, most data used in this study are before the pandemic, while the comparison between pre-pandemic and post-pandemic need further exploration in the future.

## Data Availability

The original contributions presented in the study are included in the article/supplementary material, further inquiries can be directed to the corresponding author/s.

## Footnotes

^1^ https://data.sh.gov.cn/
